# Creosote and Creosotal in Pneumonia

**Published:** 1903-02

**Authors:** 


### ABSTRACTS.

### Creosote and Creosotal in Pneumonia.

Last March, Dr. I. L. Van Zandt, of Fort Worth, Texas, (*Medical Record*, October 11, 1902,) sent out a number of circulars to many medical journals and to a few individuals, asking the following questions of those who had used creosote or carbonate of creosote (creosotal) in the treatment of pneumonia: (1) Do you believe creosote ever aborts pneumonia? (2) Do you believe the majority of cases are mitigated by it? (3) Have you ever found cases which, having plenty of time, were entirely uninfluenced by it?

In response he had over seventy letters and cards and five verbal statements, a large proportion of which he tabulated. To the first question 37 physicians, reporting 762 cases, said "yes;" 15, reporting 187, said "no;" and 19, reporting 177, failed to answer. Therefore, of those reporting, a little over two-thirds admitted the abortive effects of creosote. To the second question, 57, reporting 1,022 cases, answered "yes;" 2, reporting 10 cases, said "no," and the remainder failed to answer. To the third, 23 said "yes;" 31, "no;" and 16 failed to answer.

Of 1,130 cases reported, 56 were fatal, 24 of them being accounted for as follows: 12 were complicated, 9 others were over the age of 67 (in some instances complicated), 3 were alcoholic (of which two were complicated); 1 was far advanced when treatment was begun, and 1 used "creosote products." The mortality in this series is a little over 5 per cent; and, as the recognised death-rate is 25 per cent., the author claims that the treatment saved 226 lives.

Van Zandt refers particularly to Prof. W. H. Thomson's report of cases treated with carbonate of creosote in the Roosevelt Hospital. The loss here was 1 in 13, or about 5.5 per cent. A condensed report for five years from this institution gives an average death-rate of 35.6 per cent.

These figures confirm the conclusions of his former article, that a large per cent. of pneumonia is cut short or aborted; almost all the rest mitigated, and the remainder or a very small per cent. not affected by the remedy. He thinks the use of creosote or carbonate of creosote in the treatment of pulmonary affections is one of the greatest life-saving discoveries of the 19th century.

